# Embolisation of the parenchymal tract after percutaneous portal vein catheterization: a retrospective comparison of outcomes with different techniques in two centres

**DOI:** 10.1186/s42155-022-00321-2

**Published:** 2022-09-05

**Authors:** Paolo Marra, Francesco Saverio Carbone, Luigi Augello, Ludovico Dulcetta, Riccardo Muglia, Pietro Andrea Bonaffini, Angelo Della Corte, Stephanie Steidler, Simone Gusmini, Giorgia Guazzarotti, Diego Palumbo, Massimo Venturini, Francesco De Cobelli, Sandro Sironi

**Affiliations:** 1 Department of Radiology, ASST Papa Giovanni XXIII Hospital, Piazza Oms 1, 24127 Bergamo, Italy; 2 School of Medicine, University of Milano–Bicocca, Piazza dell’Ateneo Nuovo, 1, Milan, Italy; 3 Department of Radiology, Vita–Salute San Raffaele University Hospital, Via Olgettina, 60, Milan, Italy; 4 School of Medicine, Vita–Salute San Raffaele University, Via Olgettina 58, Milan, Italy; 5 Department of Radiology, Circolo Hospital, Macchi Foundation, Insubria University, Via Luigi Borri, 57, Varese, Italy

**Keywords:** Percutaneous portal vein catheterization, Parenchymal tract embolisation, Glue, Gelfoam, Coils

## Abstract

**Background:**

Embolisation of the parenchymal tract is a key step after any other transhepatic or transplenic percutaneous portal vein catheterization since eventual venous bleeding is difficult to control and may require surgical management. Different techniques have been proposed to perform tract embolisation. The aim of this study is to compare the safety and efficacy of different techniques of haemostasis of the parenchymal tract.

**Materials and methods:**

All the interventional procedures with percutaneous transhepatic or transplenic access to the portal vein (excluding ipsilateral portal vein embolisation) from January 2010 to July 2020, in two tertiary hospitals, were retrospectively analyzed. The following data were evaluated: access site, the technique of embolisation, technical success in terms of immediate thrombosis of the tract, safety and clinical efficacy in terms of the absence of hemorrhagic and thrombotic complications.

**Results:**

One-hundred-sixty-one patients underwent 220 percutaneous transhepatic or transplenic portal vein catheterization procedures. The main indications were pancreatic islet transplantation, portal anastomotic stenosis after liver transplantation, and portal vein thrombosis recanalization. As embolic materials gelfoam was used in 105 cases, metallic micro-coils in 54 cases, and cyanoacrylic glue in 44 cases; in 17 cases the parenchymal tract was not embolized. Technical success was 98% without significant difference among groups (*p*-value = 0.22). Eighteen post-procedural abdominal bleedings occurred, all grade 3 and were managed conservatively; difference among groups was not significant (*p*-value = 0.25). We detected 12 intrahepatic portal branch thromboses not related to the embolisation technique; only one case of non-target embolisation was documented after liver tract embolisation with glue, without clinical consequences.

**Conclusion:**

Embolisation of the parenchymal tract after percutaneous portal vein catheterization is technically safe and effective. No significant differences were found between coils, glue, and gelfoam in effectiveness and complications rate.

**Level of evidence:**

Level 3, Cohort study.

## Background

The indications for percutaneous portal vein catheterization (PPVC) have significantly grown in recent years, due to technical developments of interventional radiology and organ transplantation (Saad and Madoff 2012; Ohm et al. 2017). Transhepatic and transplenic portal vein accesses are mainly performed to manage complications of liver transplant, such as stenosis and thrombosis, to induce liver hypertrophy before major resections, with portal vein embolisation, and to administer autologous or heterologous cells in the setting of pancreatic islet transplantation (Saad and Madoff 2012; Denys et al. 2012; Venturini et al. 2018). Although the issue of haemostasis is considered less relevant during portal vein embolisation with the ipsilateral approach (Saad and Madoff 2012), the embolisation of the parenchymal tract should be regarded as a key step after any other transhepatic or transplenic PPVC, since eventual venous bleeding is difficult to control and may require surgical management.

Different techniques have been proposed to perform tract embolisation and their use varies according to the operator experience: gelfoam, cyanoacrylates, coils, and plugs are among the most used materials (Saad and Madoff 2012; Dollinger et al. 2013).

Several authors have already reported the safety and efficacy of parenchymal tract embolisation with coils, glue, or a combination of both in after a transhepatic or transplenic approach. (Ohm et al. 2017; Zhang et al. 2021; Park et al. 2014; Haddad et al. 2018; Zhu et al. 2013; Chu et al. 2012). The use of gelfoam was reported to be safe and effective in pediatric patients with orthotopic liver transplant (Uller et al. 2014). To our knowledge no consensus exists about the most effective and safe embolic agent nor if the tract embolisation should be considered necessary.

The aim of the present study is to report the retrospective data of two tertiary referral centres that commonly perform PPVC, to compare the technical and clinical outcomes of a non-operative management in obtaining haemostasis of the portal vein access and the outcomes of the use of different embolic materials to perform parenchymal tract embolisation.

## Materials and methods

### Study design

This is a retrospective cohort study in patients who underwent transhepatic or transplenic PPVC procedures between January 2010 and December 2020 in two tertiary referral centres for liver and pancreatic surgery and transplantation. Patients were identified using case match search words from Institutional radiological records. Written informed consent for the interventional procedures was obtained prior to treatment from all the patients and/or from legal guardians in case of minors. Local Ethical Committees authorized this retrospective study that was conducted in respect of the principles of the Declaration of Helsinki.

Inclusion criteria were:

- a radiological procedure of percutaneous portal vein catheterization, both transhepatic of transplenic;
- availability of procedural technical details in interventional radiology reports;
- availability of periprocedural imaging for review: at least an Ultrasound (US) or Computed Tomography (CT) examination 1 week before and up to 1 month after the interventional procedure;
- availability of 1-month post-procedural clinical data.

Exclusion criteria were:

- PPVC for portal vein embolisation with ipsilateral approach;
- absence of procedural technical details in interventional radiology reports;
- absence of periprocedural imaging and clinical follow-up;
- tract embolisation performed with unusual devices, such as vascular plugs, or a combination of materials.

Early post-procedural imaging was not routinely performed unless upon clinical indication in case of uncontrolled pain, suspected bleeding, or organ dysfunction.

### Study endpoints and outcome measures

The primary objective was the technical success of the techniques, defined as the intraoperative evidence of release of embolic materials in parenchymal tract and/or the ability to remove the percutaneous sheath without any signs of bleeding.

The secondary endpoint was a clinical success in terms of the absence of post-procedural bleeding and the absence of non-target embolic complications. Post-procedural imaging bleeding was defined by the development of acute pain, a drop of hemoglobin level, and/or hemodynamic instability from 1 hour to 72-hours after the procedure, confirmed by the evidence of abdominal fluid collections or hematomas at imaging. Non-target embolisation was assessed by any available post-procedural imaging study up to 1 month (or until further interventions were performed) and it was defined as the evidence of embolic material inside the vessel lumen. Intrahepatic portal vein thrombosis detected at post-procedural imaging was also recorded, regardless of evidence of non-target embolisation.

Bleeding, non-target embolisation, and thrombotic events were considered procedure-related complications and were graded according to the CIRSE Quality Assurance Document and Standards for Classification of Complications (Filippiadis et al. 2017).

### PPVC technique

All the patients were considered eligible for PPVC if no medical or technical contraindications were present. The procedures were performed electively or deferred where possible depending on clinical urgency. All available procedure data were collected. Coagulopathy (International Normalized Ratio ≥ 2 and platelet count < 50.000) and perihepatic or perisplenic ascites were considered absolute contraindications, unless corrected with transfusions or drained, respectively.

In Centre 1, the main indication for PPVC was portal vein stenosis or thrombosis in liver-transplanted patients. The choice of interventional approach was based on imaging findings, at the discretion of the interventional radiologist based on experience; the primary approach was via a transhepatic route with a transplenic approach used as the second choice if this was not feasible. In Centre 2, the main indication was pancreatic islet transplantation.

All procedures in both centres were performed using fluoroscopy and digital subtraction angiography in the angiographic suite; both centres were equipped with Allura Xper FD20 (Philips Healthcare, Best, the Netherlands). Interventional radiologists had at least 5 years of experience. Conscious sedation with midazolam and fentanyl was used for adults, while general anesthesia was performed by a dedicated anesthesiologist in paediatric patients (after induction with propofol, fentanyl, and rocuronium bromide, general anesthesia was maintained with sevoflurane).

PPVC technique was the same in both centres: a parenchymal branch of the portal vein system was punctured under US guidance with a 22G Chiba needle; the guidewire was then advanced under fluoroscopic guidance and a standard coaxial 4F introducer system (Neff percutaneous access set, Cook Incorporated, Bloomington, IN; AccuStick, Boston Scientific, Marlborough, MA) was pushed over the guide. For pancreatic islet transplantation, cell infusion was performed through the 4F introducer sheath; for all the other procedures that required portal vein navigation up to 7F standard vascular introducer sheaths (Merit Medical System, South Jordan, UT) were used. In case of portal vein stenosis or thrombosis, sodium heparin was transcatheter administered in the portal vein with boluses ranging from 50 to 80 units/kg.

The study population was divided into four groups, regardless of indication, based on the embolisation technique selected by the interventional radiologist during the procedure:

- Group 1: cyanoacrylate and ethiodized oil (Glubran 2, GEM, Viareggio, Italy; Lipiodol, Guerbet, Villepinte, France);
- Group 2: coils 2–3 mm of diameter × 30 mm of length (MReye, Cook Incorporated, Bloomington, IN);
- Group 3: gelfoam torpedoes (Johnson & Johnson Medical N.V., Belgium);
- Group 4: no tract embolisation performed.

**Fig. 1:**
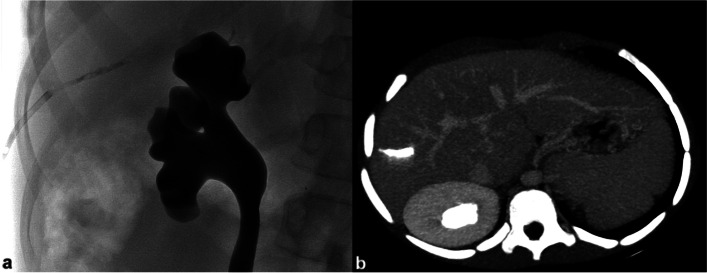
Embolisation of transhepatic tract with cyanoacrylic glue after PPVC. Fluoroscopy image shows the deployment of cyanoacrylic glue in the hepatic parenchyma tract, through a 4F introducer sheath during its retraction (**a**). MIP reconstruction of contrast-enhanced CT performed after the procedure displays the location of glue cast in the hepatic parenchyma and confirms the adjacent segmental portal branches patency (**b**)

**Fig. 2:**
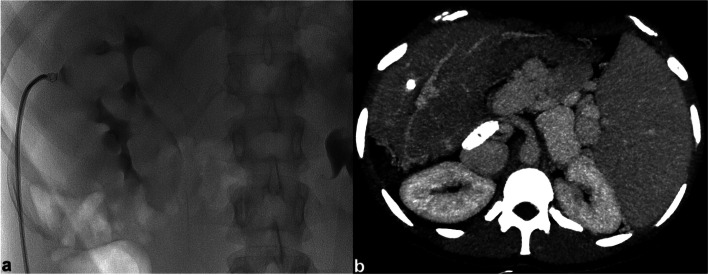
Embolisation of transhepatic tract with micro-coil after PPVC. Fluoroscopy image shows the deployment of a metallic micro-coil in the hepatic parenchyma tract, through a 4F catheter during its retraction (**a**). MIP reconstruction of contrast-enhanced CT performed after the procedure displays the location of micro-coil in the hepatic parenchyma and confirms the segmental portal branches patency (**b**)

Tract embolisation with glue and gelfoam was performed through the introducer sheath during its retraction (Saad and Madoff 2012); coil embolisation was performed through standard hydrophilic 4F catheters (Terumo Corporation, Tokyo, Japan; Cordis Corporation, Miami Lakes, FL). Small contrast boluses verified positioning of the introducer or catheter tip as it pulled back from the vessel lumen into the parenchyma (Saad and Madoff 2012); after flushing with dextrose 5%, 0.1–0.2 ml of 1:2 cyanoacrylate/Lipiodol mixture were injected under fluoroscopic guidance (Fig. 1). In groups 2 and 3, coils or gelfoam torpedoes were pushed with the stiff end of a 0.035″ hydrophilic guidewire (Terumo J; Terumo Corporation, Tokyo, Japan), until disappearance of blood reflux (Fig. 2). In all cases, following embolisation, manual compression was applied for 1–2 minutes. When no tract embolisation was performed, the introducer sheath was gradually pulled back and removed upon the disappearance of blood reflux; manual compression was then applied for 10 minutes.

### Statistical analysis

Continuous data are presented as the medians and interquartile range (IQR), categorical data as numerical values and percentages. Descriptive and analytic statistics were calculated using IBM SPSS Statistics Version 26.0 (IBM Corp., Armonk, NY). Chi-squared test and Fisher’s exact test were applied to contingency tables with the Bonferroni corrected alpha level for post-hoc pairwise comparisons.

## Results

### Study population and PPVC characteristics

**Fig. 3:**
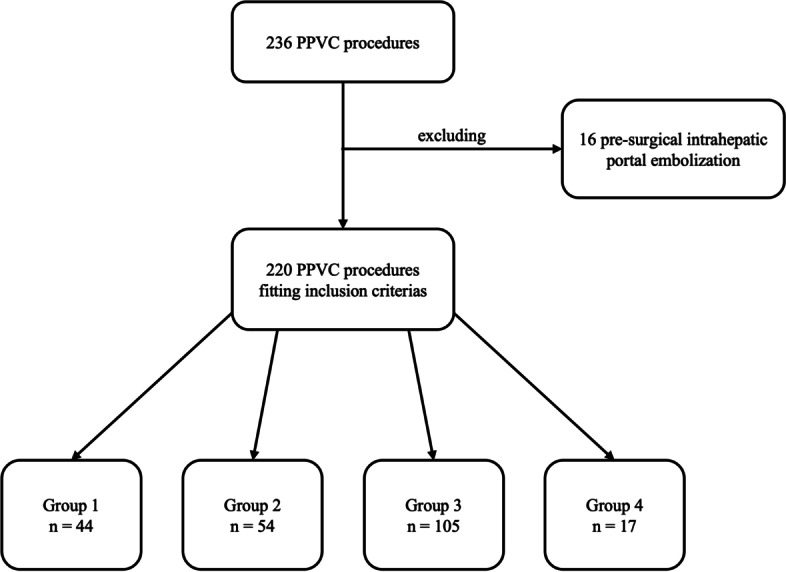
Flow diagram summarizes the population selection

Extracted data that met inclusion criteria included 220 PPVC procedures performed in 161 patients (median age = 35.7 years, IQR = 40.7 years; 80 females) (Fig. 3). The mean number of procedures per patient was 1.4 (range 1–6); 43 patients underwent more than one PPVC. Seventy-seven (35%) PPVCs were performed in paediatric patients, and 74 (34%) procedures were performed in orthotopic transplanted livers. Patient characteristics, indications for PPVC, and portal access features for each group are summarized in Table 1.

**Table 1:** Patient and PPVC baseline characteristics

***Characteristics***	***Group 1***	***Group 2***	***Group 3***	***Group 4***
	*n* = 44	*n* = 54	*n* = 105	*n* = 17
***Sex***				
*Female (%)*	22 (50)	28 (52)	55 (52)	10 (59)
*Male (%)*	22 (50)	26 (48)	50 (48)	7 (41)
***Age****, years*				
*Median [IQR]*	13.1 [36.1]	15.9 [47.6]	44.6 [20.4]	10.9 [38.2]
***Indication to PPVC***				
*Pancreatic islet transplantation (%)*	0 (0)	0 (0)	104 (99)	2 (12)
*Portal anastomosis stenosis in OLT (%)*	16 (36)	40 (74)	0 (0)	10 (59)
*Portal vein stenosis/thrombosis (%)*	7 (16)	7 (13)	0 (0)	1 (6)
*TIPS creation (%)*	15 (34)	3 (6)	1 (1)	3 (18)
*Meso-rex anastomosis stenosis (%)*	3 (7)	4 (7)	0 (0)	1 (6)
*Portal varices embolisation (%)*	3 (7)	0 (0)	0 (0)	0 (0)
***Type of approach for PPVC***				
*Right transhepatic (%)*	14 (32)	26 (48)	101 (96)	7 (41)
*Left transhepatic (%)*	5 (11)	6 (11)	4 (4)	1 (6)
*Lateral left split liver (%)*	18 (41)	19 (35)	0 (0)	7 (41)
*Main portal trunk (%)*	0 (0)	0 (0)	0 (0)	2 (12)
*Trans-splenic (%)*	7 (16)	3 (6)	0 (0)	0 (0)
***Internal calibre of PPVC sheath****, French*				
*Median*	6	6	4	6

*IQR* Interquartile range, *PPVC* Percutaneous portal vein catheterization, *OLT* Orthotopic liver transplantation, *TIPS* Trans-jugular intrahepatic portosystemic shunt

At baseline, in Group 1, Group2, and Group 4 the main indication for PPVC was portal vein stenosis or thrombosis in liver transplants. In Group 3, the indication for PPVC was pancreatic islet transplantation in all but one case, with a significantly higher median age in this Group (*p*-value < 0.001). One-third (*n* = 15) of patients in Group 1 underwent PPVC in combination with trans-jugular intrahepatic portosystemic shunt (TIPS) creation.

The most common approach to the portal vein was transhepatic (*n* = 210/220; 95%), through the right hepatic lobe segmental branches in most cases (148/210; 70%). In paediatric patients with transplanted left lateral segments (*n* = 40), a segment 3 portal branch access was performed. Paediatric patients with split grafts were present in all groups, but not in Group 3 (*n* = 13 in Group 1; *n* = 17 in Group 2; *n* = 5 in Group 4). Transplenic PPVC was performed in 10 procedures, 7 in Group 1 and 3 in Group 2.

The size of the introducer sheath was significantly smaller in Group 3 than in the other groups (median 4 F versus 6 F; *p*-value < 0.001).

### Technical success and clinical outcomes

Technical success and clinical outcomes of the procedures are summarized in Table 2. Technical failure was 6% (*p*-value = 0.22) when tract embolisation was not performed (Group 4). In all the other groups technical success was higher, without statistically significant differences (Groups 1 and 2 = 98%; Group 3 = 100%).

**Table 2:** Procedural technical and clinical success

	***Group 1***	***Group 2***	***Group 3***	***Group 4***	***p-value***
	*n* = 44	*n* = 54	*n* = 105	*n* = 17	
***Procedural technical success*** *(%)*	43 (98)	53 (98)	105 (100)	16 (94)	0.22
***Procedural clinical success (%)***	1 (2)	7 (13)	8 (8)	2 (12)	0.26
***Complications***					
***Intrahepatic portal vein thrombosis*** *(%)*	3 (7)	4 (7)	1 (1)	4 (24)	0.001
***Non-target embolisation*** *(%)*	1 (2)	0 (0)	0 (0)	NA	0.26

*NA* Not applicable

A total of 18/220 (8%) symptomatic bleeding episodes were reported; in all cases, clinical suspicion and imaging confirmation occurred within the first 24 hours after the interventional procedure, with a higher incidence in Group 2 and Group 4, not reaching statistical significance (*p*-value = 0.25). Intra-procedural anticoagulants were administered only in patients in Group 1 and 2, all with no bleeding events reported. A larger introducer sheath size that was more common in groups 1, 2 and 4 was not associated with technical failure nor bleeding events.

Only one case of non-target embolisation in peripheral segmental portal vein branches was documented after transhepatic tract closure with glue (Group 1), without clinical consequences.

A total of 12 (6%) cases of portal vein thrombosis were detected at imaging follow-up, with a significantly higher prevalence in Group 4 (*p*-value = 0.001).

Comparable technical success rates and favorable clinical outcomes were observed among the transhepatic and the transplenic approaches.

No grade 1 or 2 complications were observed. All bleeding, non-target embolisation, and thrombotic events were scored grade 3 and conservatively managed. No complications with clinical sequelae were reported.

## Discussion

In this study, data collected over 10 years from two large tertiary liver centres specialized in diabetes, pancreatic surgery, and liver transplantation which commonly perform PPVC, was retrospectively evaluated. Standard techniques for parenchymal tract haemostasis were compared, considering clinical indications for PPVC and technical outcomes. The haemostasis technique was selected independently by the interventional radiologist and it was based on experience and evaluation of bleeding risk. The retrospective nature of this study over a long period made it difficult to collect more procedural details (including anticoagulation therapy) and peri-procedural clinical data.

Tract embolisation with all the devices included in this cohort was technically and clinically successful, without statistical differences among groups. As expected, a slightly higher technical failure rate was observed when no tract embolisation was performed, although without statistical difference. Concerning post-procedural bleeding, it symptomatically occurred in 8% of cases in the first 72-hours after the procedure, which is far below the reported rate of up to 30% (Saad and Madoff 2012). This means that overall post-procedural haemostasis was effective. No statistically significant differences were found between embolisation techniques in the post-procedural bleeding rate, nor when haemostasis was performed with manual compression. The lower incidence of post-procedural bleeding in this cohort was obtained when embolisation was performed with glue. However, the difference didn’t reach statistical significance. In all cases, technical failures and bleeding events did not determine relevant clinical sequelae, and patients were conservatively managed. The efficacy of glue for parenchymal tract haemostasis after PPVC has been already shown by several authors: Zhang et al. (2021) in a single-centre retrospective comparison of glue and coils for PPVC tract embolisation presented similar results. They observed a reduced rate of bleeding events when embolisation was performed with glue, at the expense of an increased, though negligible, risk of non-target occlusion of intrahepatic portal branches. The authors reported the presence of fewer artifacts with glue at CT imaging, which is an advantage in patients undergoing routine CT follow-up. Also, Park et al. (2014) reported favorable outcomes of tract embolisation performed with glue. Our data corroborate these findings.

Concerning post-procedural thrombotic events, they were observed in a small percentage of cases, only in one determined by non-target embolisation with glue, without clinical consequences. Data from other studies (Zhang et al. 2021; Park et al. 2014) confirm our findings, with a low incidence of non-target embolisation using cyanoacrylate glue, when performed with a proper technique. Only few distal portal branches were involved in case of non-target embolisation, without any subsequent clinical signs of infection or ischemia, as similarly reported by Zhang et al. (2021). A significantly higher incidence of intrahepatic portal branches thrombosis was detected when the embolisation of the parenchymal tract was not performed; this may be explained by the fact that thrombosis was already present before PPVC and the interventional radiologist did not considered necessary to perform tract embolisation. Even though data confirms the low risk of non-target embolisation, the technical difficulty of the procedure and the expertise of the interventional radiologist always need to be considered.

Despite a lower prevalence of transplenic approaches in the study cohort, no differences were found in terms of technical and clinical outcomes of the different modalities of tract embolisation. Haddad et al. (2018) and Ohm et al. (2017) showed safety and efficacy of coils and a combination of glue and coils, respectively, and Zhu et al. (2013) also described successful parenchymal tract embolisation using glue in a cohort of patients treated with transplenic portal vein interventions. Chu et al. (2012) reported the use of a combination of coils and a mixture of lipiodol and glue without bleeding complications in a small cohort of patients treated with a transplenic approach. Overall, these findings indicate the splenic vein as a safe route for percutaneous portal vein interventions.

Of note, in this study the combination of different embolic materials was not considered, in order to avoid technical bias for the heterogeneity of procedural embolisation (e.g., different proportion in different materials). Although, the combination of coils and glue is reported to be effective, we consider it unnecessary and costly: indeed, we obtain an adequate haemostasis even when glue was used alone. We suggest using a Lipiodol/glue ratio lower that 3 to obtain rapid polymerization and prevent non-target embolisation. The performance of other devices proposed for PVCC, in particular, plugs (Dollinger et al. 2013), vascular closure kits (Tan et al. 2020), and microfibrillar collagen paste (Gaba et al. 2017), was not assessed, as was the recently described PVCC via the mesenteric vein (Onishi et al. 2021).

The study cohort includes a large number of paediatric patients with orthotopic liver transplant in which both transhepatic and exceptional transplenic accesses were used. Parenchymal tract embolisation in these patients was successfully performed with coils, glue and in few isolated cases, not performed. Available data in the literature reports safe and effective use of gelfoam in this sub-group of patients (Uller et al. 2014); to our knowledge, very few cases of tract embolization with coils and vascular plugs are reported in paediatric patients (Dollinger et al. 2013; Chu et al. 2012).

The different indications for PPVC in this study may also have relevant clinical implications. Transplanted patients with portal vein stenosis or thrombosis usually present with portal hypertension, or a hyperdynamic circulation after a successful portal recanalization, which may increase the propensity to bleeding. Furthermore, they normally receive perioperative anticoagulation. On the contrary, patients undergoing pancreatic islet transplantation rarely suffer from portal hypertension and the portal pressure increase after islet injection is negligible (Venturini et al. 2018); moreover, they do not receive perioperative anticoagulation. In liver transplanted patients with portal vein obstruction the introducer sheath ranges up to 7F while in pancreatic islet transplanted patients it is 4F at maximum. All these factors determine a theoretical higher risk of bleeding in liver-transplanted patients. Nevertheless, bleeding events occurred in both clinical situations, without statistically significant differences, but with a trend towards more bleeding events in the lower risk population. We think this is due to the more effective hemostatic potential of glue, compared to gelfoam.

## Conclusion

The outcomes of this retrospective study in conjunction with the literature supports the conclusion that all proposed embolization methods are valid. The embolization of the parenchymal tract after PPVC is a safe and effective procedure, useful to limit postprocedural bleeding. Based on a multicenter experience, we lean towards the use of cyanoacrylic glue for the embolisation of higher calibre trans-parenchymal accesses, which is supported by a good control of hemostasis without complications compared with other materials. However, given that the difference in terms of technical success and clinical outcomes among embolization techniques did not reach statistical significance, the choice of the embolization technique could be based on the operator’s preferences and experience. The large heterogeneity of clinical conditions, indications to PPVC, access calibre and types among the groups of patients considered, and the two centres, as well as the number of interventional radiologists performing the procedure, introduce some bias to the results. The demonstration of safety of all methods supports the potential for a prospective study of the subject, which could include a cost-benefit analysis.

## Data Availability

The datasets used and/or analyzed during the current study are available from the corresponding author on reasonable request.

## Abbreviations

PPVC: Percutaneous portal vein catheterization
US: Ultrasound
CT: Computed tomography
IQR: Interquartile range
TIPS: Transjugular intrahepatic portosystemic shunt
OLT: Orthotopic liver transplantation
NA: Not applicable
